# Behavioral preventive measures and the use of medicines and herbal products among the public in response to Covid-19 in Bangladesh: A cross-sectional study

**DOI:** 10.1371/journal.pone.0243706

**Published:** 2020-12-11

**Authors:** Iftekhar Ahmed, Maruf Hasan, Rahima Akter, Bidduth Kumar Sarkar, Marufa Rahman, Md Samun Sarker, Mohammed A. Samad

**Affiliations:** 1 Department of Pharmacy, Jahangirnagar University, Savar, Dhaka, Bangladesh; 2 Department of Pharmacy, World University of Bangladesh, Dhaka, Bangladesh; 3 Department of Pharmacy, Ranada Prasad Shaha University, Narayanganj, Bangladesh; 4 Antimicrobial Resistance Action Centre (ARAC), Bangladesh Livestock Research Institute (BLRI), Savar, Dhaka, Bangladesh; University of Dhaka, BANGLADESH

## Abstract

The present study was conducted to assess the behavioral preventive measures and the use of medicines and herbal foods/products among the public in response to Covid-19. A cross-sectional survey comprised of 1222 participants was conducted from 27 June to 20 July 2020. Kruskal-Wallis test was used to identify the differences in behavioral preventive practices across different demographic categories. To identify the factors associated with the use of preventive medicines and herbal foods/products, multivariable logistic regression was performed. Most participants adopted the recommended preventive practices such as washing hands more frequently (87.5%), staying home more often (85.5%), avoiding crowds (86%), and wearing masks (91.6%). About half of the smokers reported a decreased rate of smoking during the pandemic. Also, 14.8% took medicines, 57.6% took herbal foods/products, and 11.2% took both medicines and herbal foods/products as preventive measure against Covid-19. Arsenicum album, vitamin supplements, and zinc supplements were the most commonly used preventive medicines. Gender, age, and fear of Covid-19 were significantly associated with the use of both preventive medicines and herbal foods/products. For the management of Covid-19 related symptoms, paracetamol, antihistamines, antibiotics, and mineral (zinc and calcium) supplements were used most often. Most participants sought information from non-medical sources while using medicines and herbal products. Moreover, potentially inappropriate and unnecessary use of certain drugs was identified.

## Introduction

Covid-19 is an infectious disease caused by a novel coronavirus named Severe acute respiratory syndrome coronavirus 2 (SARS-CoV-2). First identified in December 2019 in China, Covid-19 was declared a pandemic in March 2020 by the World Health Organization (WHO) [1]. Bangladesh is one of the most affected countries by the Covid-19 pandemic, with 232,194 confirmed cases as of 30 July 2020 [2]. Bangladesh is a country with limited resources and a poor healthcare management system. The country is facing a multitude of challenges to fight this pandemic including insufficient testing, lack of public awareness, and inadequate facilities to treat Covid-19 patients [3, 4]. For instance, the capability of the government to treat Covid-19 patients is extremely limited, with only 733 intensive care unit (ICU) beds and fewer than 1,800 ventilators for critical support for a population of well over 16 million [5]. There have been reports of Covid-19 patients being denied treatments from public and private hospitals as these hospitals do not have the required facilities to treat Covid patients [5, 6].

Given the situation, hospital-based interventions cannot be a primary means of dealing with the pandemic in resource-limited countries like Bangladesh. Instead, efforts must be made to prevent the spread of the virus as much as possible. Recommended preventive measures include wearing masks, social distancing, washing hands regularly, and staying home as much as possible [7]. In absence of vaccines or antiviral therapy against Covid-19, experts have recommended the intake of vitamins, minerals, and herbal medicines in order to bolster the immune system in a bid to lower the risk and severity of infection [8–10].

The panic and fear surrounding the pandemic combined with misinformation have prompted the people to buy and hoard medicines [11, 12]. During the Covid-19 lockdown, access to health providers has been restricted significantly which is likely to make people more prone to self-medication and more dependent on less reliable sources such as social and digital media for medicine-related information. Therefore, it is very likely that there have been inappropriate and unnecessary uses of medicines by the Bangladeshi people in relation to the prevention and cure of Covid-19 symptoms.

The primary focus of this study was to explore what medicines and herbal products/foods are being used by the public as preventive measure against Covid-19, as well as to manage symptoms associated with Covid-19. To our best knowledge, this is the first study in any country to assess the use of medications among the public in response to Covid-19. We also assessed the behavioral preventive measures adopted by the public during the pandemic.

## Methods

### Study design and sampling

We performed a cross-sectional survey from 27 June to 20 July 2020 using a self-administered questionnaire. Data collection was conducted both in-person and online using purposive sampling to include participants of all ages and diverse educational and socioeconomic backgrounds. The online survey was conducted using google forms and participants were recruited via social media platforms such as Facebook and WhatsApp. Since most internet users are young and live in urban areas, we also conducted in-person surveys to recruit participants from rural areas who might not have access to the internet. The in-person survey was done in different parts of the country including Dhaka, Manikganj, Cumilla, Tangail, and Nilphamari. Adults aged 18 years and above were eligible to participate in the survey. Regarding sample size, the goal was to include as many participants as possible. The research protocol was reviewed and approved by the Antimicrobial Resistance Action Center (ARAC), Animal Health Research Division, Bangladesh Livestock Research Institute (BLRI), Bangladesh (Approval no: ARAC:15/06/2020:03). Participants were made aware of the purpose of the study and were asked to provide consent before participating in the survey. During the in-person survey, written consent was obtained from the participants. And when participating online, participants were first asked if they were willing to participate in the survey and they had to click the “Yes” button before continuing to the next sections.

### The questionnaire

The questionnaire (S1 File) consisted of three parts. The first part was for sociodemographic information such as age, gender, education, marital status, place of residence (rural or urban), presence of chronic disease, as well as participants’ fear of Covid-19. The second part contained four questions related to behavioral preventive measures adopted by the people such as washing hands, staying home, avoiding crowds, and wearing masks. Participants were also asked if there had been any changes in their smoking habits during the pandemic.

The third section asked questions about the participants’ use of medicines and herbal products/foods as preventive and curative measures against Covid-19. First, participants were asked whether they had experienced any Covid-19 related symptom or not. Those who did not experience any symptoms were asked if they had taken any medicine or herbal product as preventive measure to lower the risk of infection. As for those who experienced one or more symptoms associated with Covid-19, they were asked what medicines they had used to manage those symptoms, as well as if they had taken any medicine or herbal products/foods as preventive measure before the occurrence of symptoms. It should be mentioned that we relied on participants’ self-reports of symptoms instead of Covid-19 test results since only a very small percentage of the population is being tested, with only 6,985 people per million population [2]. To help participants with the identification of Covid-19 symptoms, a list of the common symptoms of Covid-19 (fever, dry cough, tiredness, sore throat, difficulty breathing) based on the WHO guidelines was provided with the questionnaire. Participants were also asked about the source of information/advice that influenced their choice of medications and herbal products. The questionnaire was translated to Bangla and pretested in a pilot survey of 10 people, and amendments were made where necessary.

### Statistical analysis

The sociodemographic characteristics of the participants, the behavioral preventive practices, the use of medicines and herbs, and the sources of medication-related information were analyzed descriptively. The number of behavioral preventive practices (washing hands, staying home, avoiding crowds, and wearing masks) adopted by each participant was summed up and scored from 0 to 4 (0 = when none of the preventive measures was adopted, 4 = when all of the preventive measures were adopted). To identify the differences in behavioral preventive practices across different demographic categories, the Kruskal-Wallis nonparametric test was performed. The Kruskal-Wallis test was preferred over ANOVA because the data was not normally distributed. To assess the factors associated with the use of preventive medicines and herbal foods/products, multivariable binary logistic regression was performed, calculating the adjusted odds ratios (OR) with 95% confidence interval (CI). A *P*-value of less than 0.05 was considered significant. Data analysis was performed in IBM SPSS version 25.

## Results

### Participants’ characteristics

A total of 1222 people participated in the survey. The age of the participants ranged from 18 to 82 years, and the mean age was 30.77 (SD: 12.1 years). Among the respondents, 61.4% were male, 61.9% lived in urban areas, and 52.1% had received university (undergraduate and graduate) education. 13.6% were suffering from one or more chronic diseases. The fear of Covid-19 varied, with 15.6% reporting being not afraid at all while 28% said they were very afraid. Detailed characteristics of the participants are provided in Table 1.

**Table 1:** Sociodemographic characteristics of the participants.

Characteristic	Frequency (%)
**Gender**	
Male	750 (61)
Female	466 (38)
**Age**	
18–29	767 (63)
30–44	277 (23)
45–59	112 (9)
60 and greater	60 (5)
**Residence**	
Rural	461 (38)
Urban	756 (62)
**Education**	
Secondary or lower	334 (27)
Higher secondary	245 (20)
Undergraduate degree	410 (34)
Graduate degree	226 (19)
**Marital status**	
Unmarried	662 (54)
Married	559 (46)
**Presence of chronic disease**	
No	1052 (86)
Yes	166 (14)
**Fear of Covid-19**	
Not afraid	191 (16)
Somewhat/ Moderately afraid	683 (56)
Very afraid	342 (28)

### Behavioral preventive measures

**Fig 1:**
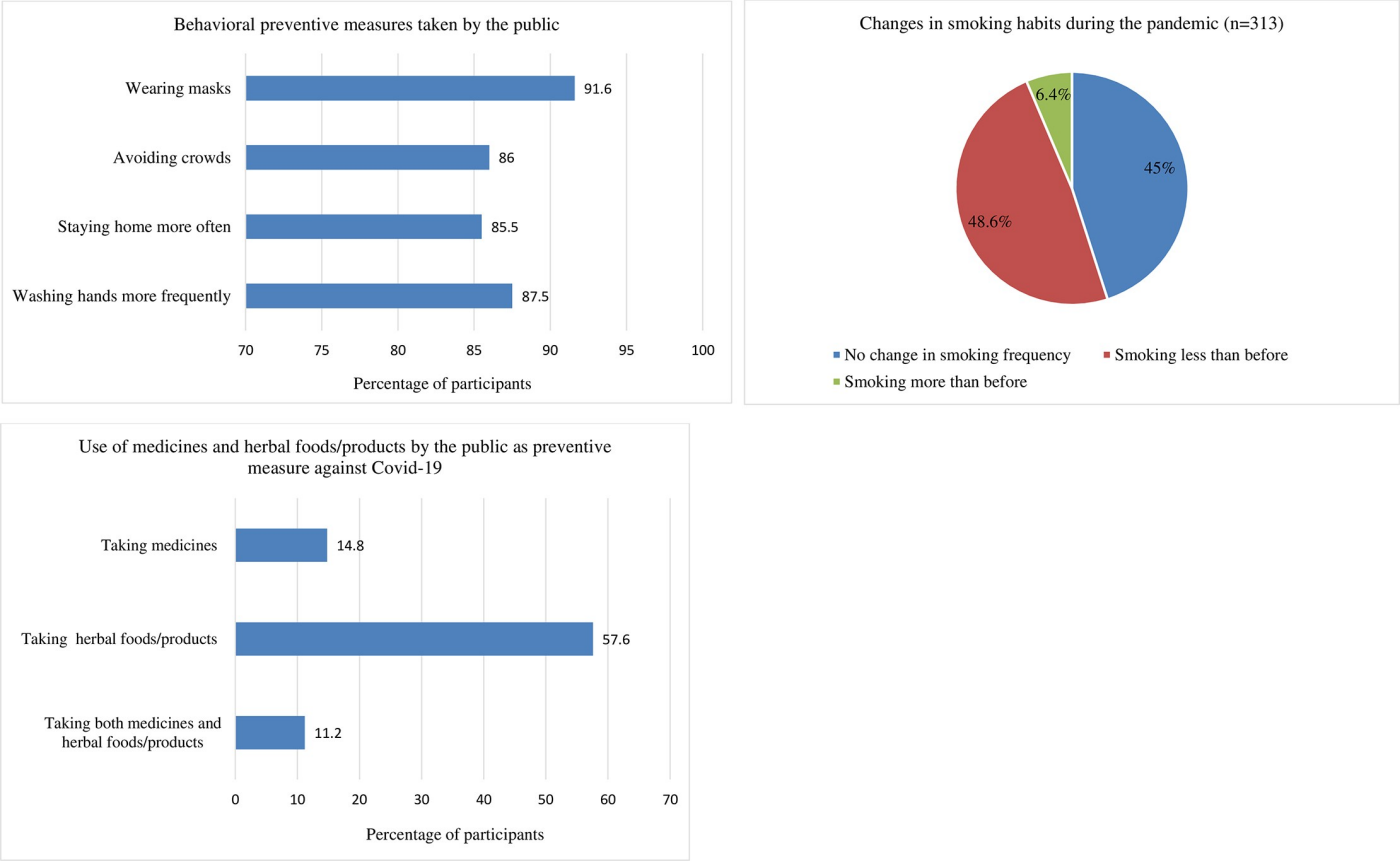
Behavioral preventive measures (A, B) and the use of preventive medicines and herbal products (C) among the public in response to Covid-19.

Most of the participants reported following the behavioral preventive guidelines such as frequent washing of hands (87.5%), staying home more often (85.5%), avoiding crowds (86%), and wearing masks (91.6%) (Fig 1A). Compliance with these practices was significantly higher among participants with higher education, participants who were females and of younger ages, and participants with a higher level of Covid-19 fear (Table 2). 25.6% (313) of the participants identified themselves as smokers, and 48.6% (152) of them said they were smoking less frequently than before the pandemic (Fig 1B).

**Table 2:** Groupwise means and the results of the Kruskal-Wallis test on the differences in the number of behavioral preventive practices across different demographic categories.

Variables	Number of preventive practices (Mean)	Standard Deviation	*P*-value
**Gender**			<0.001
Male	3.35	1.09	
Female	3.76	0.58	
**Age**			0.049
18–29	3.54	0.93	
30–44	3.48	0.95	
45–59	3.37	0.1	
60 and greater	3.37	1.06	
**Residence**			<0.001
Rural	3.25	1.17	
Urban	3.66	0.74	
**Education**			<0.001
Secondary or lower	3.00	1.25	
Higher secondary	3.62	0.85	
Undergraduate	3.71	0.71	
Graduate	3.76	0.56	
**Marital status**			0.005
Unmarried	3.57	0.89	
Married	3.43	1.0	
**Presence of chronic disease**			0.81
No	3.50	0.96	
Yes	3.52	0.87	
**Fear of Covid-19**			<0.001
Not afraid	2.76	1.42	
Somewhat/ Moderately afraid	3.57	0.81	
Very afraid	3.80	0.6	

### Medication use

**Fig 2:**
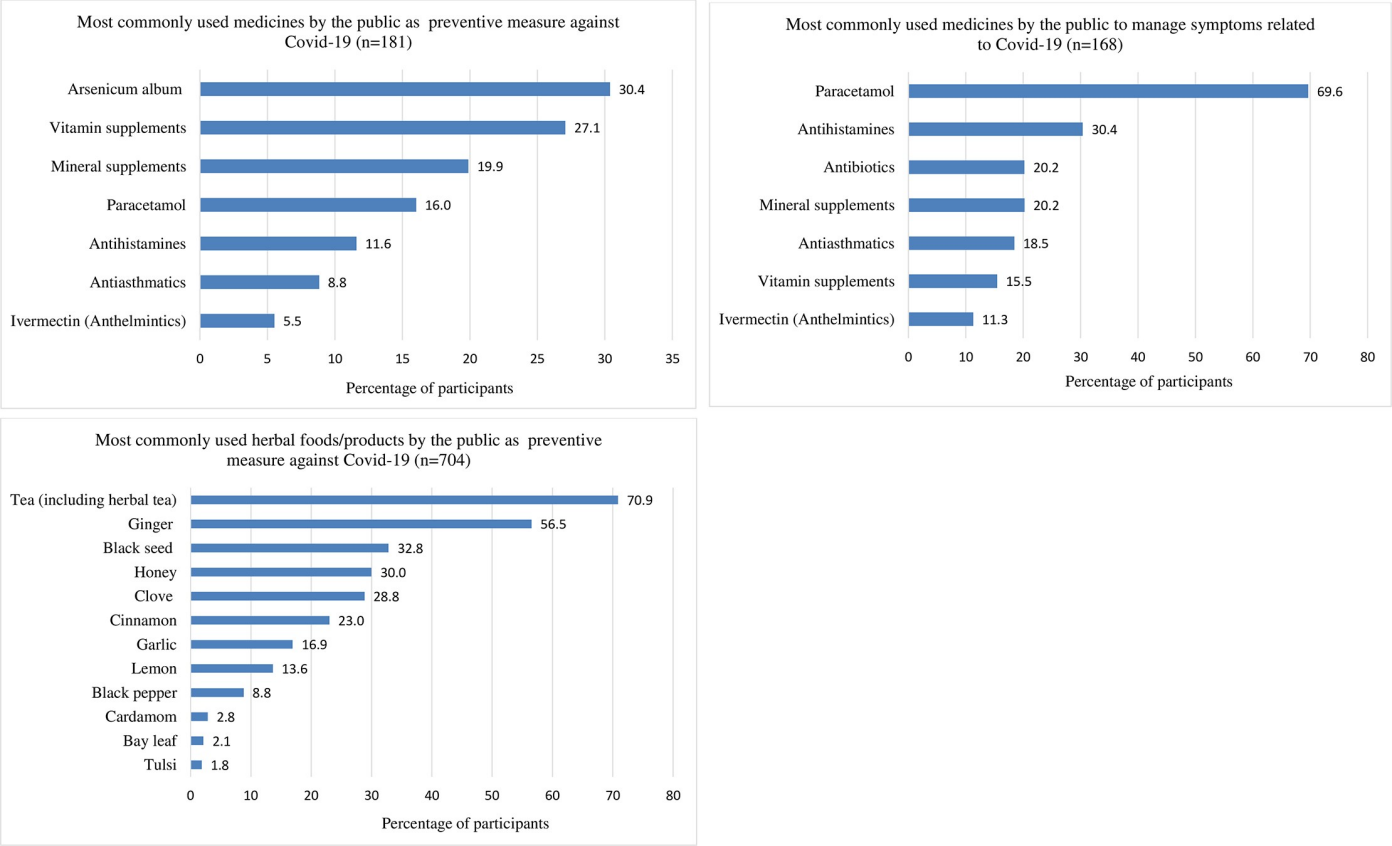
Use of medicine for prevention (A) and management (B) of Covid-19 symptoms and the use of herbal foods/products for prevention (C) of Covid-19 (the cumulative percentage may be greater than 100 since many participants took more than one drug or herb).

About 15% (181) of the participants took conventional (allopathic) and homeopathic medicines prophylactically in order to lower the risk of being infected with Covid-19 (Fig 1C). The most commonly used medicine was arsenicum album, a homeopathic formulation of arsenic trioxide. Other commonly used medicines were vitamin supplements (vitamin C, D, B, and multivitamins), mineral supplements (mostly zinc), paracetamol, antihistamines (fexofenadine, desloratadine, and chlorpheniramine), antiasthmatics (mostly montelukast), and ivermectin (Fig 2A). Results of regression analysis show that males were more likely to take preventive medicines compared to females (OR: 1.49, 95% CI: 1.04–2.15). Moreover, People aged 60 and above were most likely to take preventive medicines, and the odds of taking medicines for those who were very afraid of the pandemic was 2.48 times (95% CI: 1.36–4.53) than that of participants who were not afraid at all (Table 3).

**Table 3:** Results of multivariable logistic regression of the factors associated with the use of medicines and herbal food/products among the public as a preventive measure against Covid-19.

Variables	Use of medicines as a preventive measure against Covid-19			Use of herbal foods/ products as a preventive measure against Covid-19		
	Adjusted Odds ratios	95% Confidence interval	*P-* value	Adjusted Odds ratios	95% Confidence interval	*P-* value
**Gender**			0.03			0.016
Female	ref			ref		
Male	1.49	1.04–2.15		0.73	0.57–0.94	
**Age**			0.004			0.049
18–29	ref			ref		
30–44	2.0	1.22–3.27		0.62	0.43–0.90	
45–59	1.02	0.49–2.15		0.57	0.34–0.94	
60 and greater	2.93	1.37–6.30		0.76	0.4–1.44	
**Residence**			0.117			0.329
Rural	ref			ref		
Urban	1.36	0.93–2.0		0.87	0.66–1.15	
**Education**			0.111			0.007
Secondary or lower	ref			ref		
Higher secondary	1.43	0.84–2.45		0.91	0.62–1.32	
Undergraduate	1.89	1.13–3.16		0.82	0.57–1.19	
Graduate	1.38	0.79–2.41		1.56	1.03–2.34	
**Marital status**			0.238			0.034
Unmarried	ref			ref		
Married	1.32	0.83–2.11		1.44	1.03–2.02	
**Presence of chronic disease**			0.776			0.682
No	ref			ref		
Yes	1.07	0.66–1.73		1.08	0.75–1.56	
**Fear of Covid-19**			0.012			<0.001
Not afraid	ref			ref		
Somewhat/ Moderately afraid	1.89	1.07–3.35		2.58	1.84–3.63	
Very afraid	2.48	1.36–4.53		2.03	1.39–2.97	

ref, reference.

Around 20% (239) of the participants experienced one or more symptoms related to Covid-19 infections, and 70% (168) of them took medications to manage the symptoms. Paracetamol was the most commonly used drug, followed by antihistamines (mostly fexofenadine, desloratadine, cetirizine, and chlorpheniramine), antibiotics (mostly azithromycin, doxycycline, and amoxicillin), mineral supplements (zinc and calcium), antiasthmatics (montelukast and salbutamol), etc. (Fig 2B).

### Use of herbal foods/products

A large number of participants (57.6%) reported having taken herbal foods/products to lower the risk of Covid-19 infection (Fig 1C). About 71% of them took tea (normal and herbal), while other herbal foods such as ginger, black seed, honey, and clove were used by 56.5%, 32.8%, 30%, and 28.8% respectively (Fig 2C). These herbs were taken alone or in combination with tea or hot water. Factors significantly associated with taking herbal foods/products were being female, young (18–29 years), married, and afraid of the pandemic. Also, participants with a graduate degree were more likely to take preventive herbal foods/products than those with a secondary degree or lower (OR: 1.56, 95% CI: 1.03–2.34) (Table 3).

### Source of information related to the use of medicines and herbal foods/products

**Fig 3:**
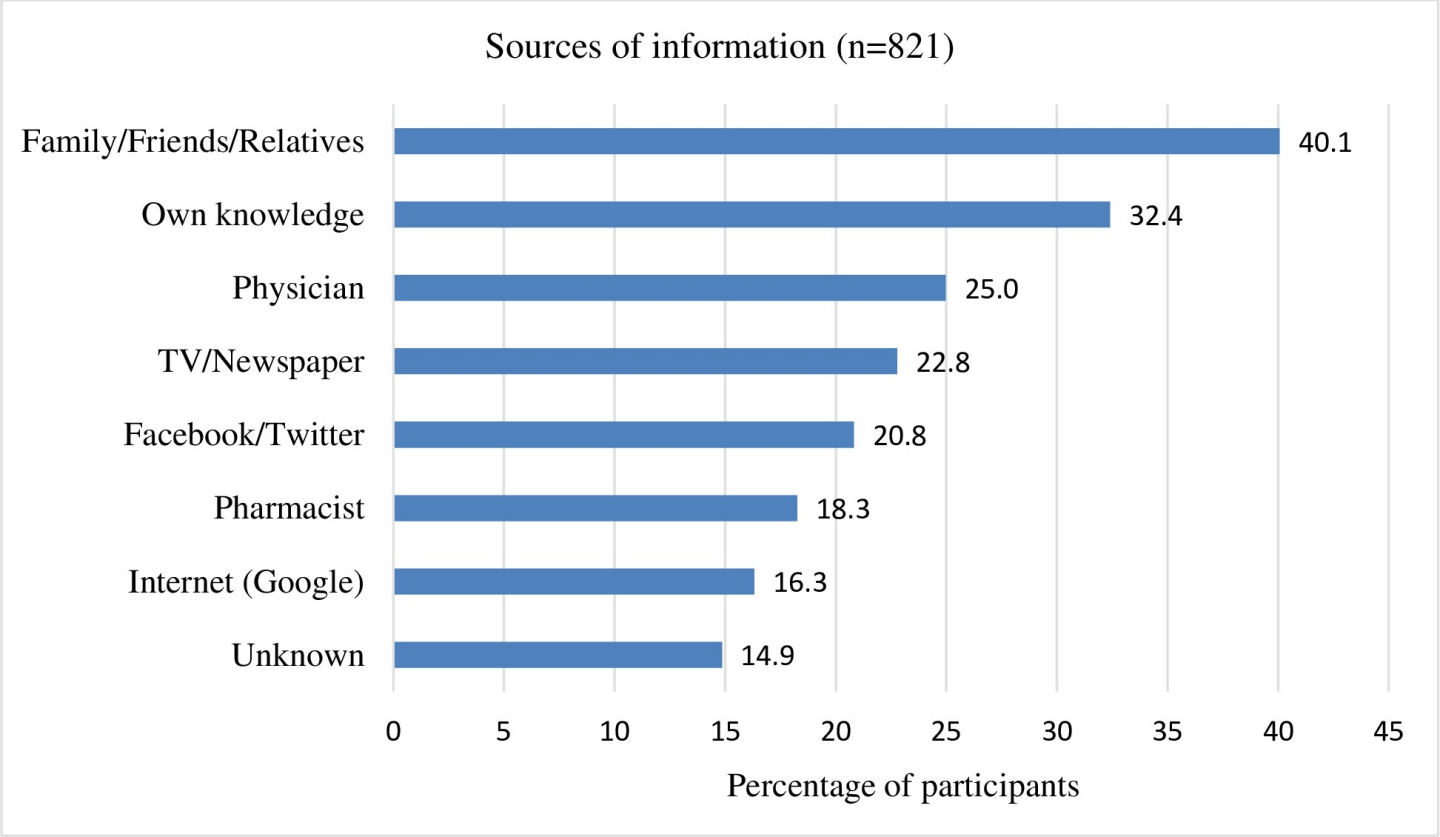
Sources of information related to the use of medicines and herbal foods/products by the public (the cumulative percentage is greater than 100 since many participants relied on more than one source of information).

When asked about the source of information related to their use of medicines or herbal foods/products, most participants said they had relied on the advice of family, friends, or relatives (40.1%). Different forms of media (print, digital, and social) were also major sources of information. Only 25% of the participants sought advice from physicians while 18% from pharmacists (Fig 3).

## Discussion

The present study was conducted at a time when the Covid-19 pandemic had reached its peak in Bangladesh [2], and we observed high rates of adoption of behavioral preventive measures (washing hands, staying home, avoiding crowds, and wearing masks), which was quite similar to the observations made in several earlier studies conducted in Bangladesh [13–15]. There was a decrease in cigarette smoking among half of the smokers. Studies have shown that smokers generally have a high risk of respiratory tract infections [16], and smoking can also be a risk factor for Covid-19 progression [17]. However, some participants reported an increase in smoking which can probably be attributed to the heightened stress, fear, and boredom associated with the pandemic and the lockdown.

A significant percentage of the participants had taken prophylactic medications, some of which were potentially unnecessary and inappropriate. For instance, the most used prophylactic medicine was the homeopathic medicine arsenicum album. Arsenicum album has been recommended as a prophylactic drug against Covid-19 by Vellingiri et al. [18], but there is no clinical evidence supporting its effectiveness against Covid-19 [19]. While there are claims that arsenicum album provides temporary relief against flu-symptoms, such claims are not proven and this drug is not currently approved by the United States Food and Drug Administration [20]. Furthermore, homeopathy in general faces lots of criticism for being implausible, unscientific, and unreliable [21, 22]. Although it is hard to tell why so many people used this drug, one of the reasons may be the fact that the Ministry of Ayurveda, Yoga & Naturopathy, Unani, Siddha and Homoeopathy (AYUSH) of the neighboring country India recommended arsenicum album for prophylactic use against Covid-19 [19]. The media may also play a role in the popularity of arsenicum album. Many participants used paracetamols prophylactically although it should only be used to manage symptoms associated with Covid-19. Another notable case was the prophylactic use of ivermectin. Out of the ten participants who used ivermectin, only two sought advice from physicians. Similar cases of ivermectin self-medication have been reported in Brazil [23]. Prophylactic use of ivermectin for Covid-19 is not only unproven, but it can also be quite risky, especially when self-medicated [23].

Since there is no effective treatment of Covid-19 at this time, the intake of vitamins, minerals, and herbs can serve to boost the immune system which can subsequently lower risks of infection and disease progression [8–10]. In this study, many participants reported having taken vitamin D, C, B, and zinc supplements. The use of herbal foods/products was also very high, probably because these are readily available in most households in Bangladesh and are known for their medicinal values. Green tea and black tea polyphenols have been reported to show antiviral activities and may have applications in Covid-19 prophylaxis and treatment [24]. Other herbal products such as ginger, garlic, honey, and black seed have also been mentioned for their antiviral actions and potential action against Covid-19 [10, 25, 26]. However, it should be mentioned that some of the reported cases of the use of herbal foods/products in this study could be habitual and participants might have taken them without being aware of their association with Covid-19 prevention (for example tea).

When investigating the drugs used for the management of symptoms associated with Covid-19, our goal was to get an overview of the commonly used medicines in the community; assessment of hospital-based treatments or management of critical patients was beyond the scope of this study. Most people infected with Covid-19 experience only mild symptoms and intake of antipyretics (paracetamol) may be sufficient for that, according to WHO [27]. The national guidelines on clinical management of Covid-19 recommend paracetamol and fexofenadine for the management of mild symptoms as well as suggest the intake of zinc and vitamin C [28]. Similar to the guidelines, in this study, paracetamol, fexofenadine, and zinc supplements have been found to be the most commonly used medicines for symptom management.

In the present study, fear of Covid-19 has been the most important factor associated with the adoption of preventive measures among the public; those who reported being afraid of Covid-19 adopted a greater number of behavioral preventive practices and were more likely to take preventive medicines and herbs. No other study has yet investigated the association between Covid-19 fear and medication use among the public, but in a study conducted in Turkey, fear of the pandemic was associated with a higher level of engagement in preventive practices [29]. Another study among the Thai healthcare workers found that those who reported fear and anxiety were more likely to adopt preventive practices such as washing hands and wearing masks and personal protective equipment [30].

While the media (television, newspaper, Facebook, Twitter) have been playing a significant role in raising awareness about the pandemic, they have also contributed to the spread of misinformation surrounding Covid-19 [31, 32]. In this study, we have found a high reliance of the participants on the media for medication-related advice related to Covid-19. Therefore, it is important that the spread of misinformation through digital, print, and social media is contained as much as possible.

### Limitations

The study has some limitations. First, the data collection was based on purposive sampling and so the findings of this study may not be generalized to the whole population. Second, we relied on self-reported data and so there is a possibility of social desirability bias where participants may prefer to give responses that are more appropriate or expected from them (like wearing mask or social distancing) instead of the responses that reflect their true behaviors.

## Conclusions

In summary, we have observed a high adoption of behavioral preventive measures by the public. Also, a considerable number of participants have taken preventive medicines and herbal foods/products. Fear of Covid-19 has been identified as one of the most important factors associated with the adoption of behavioral preventive measures as well as the use of preventive medicines and herbal foods/products. For information pertaining to the use of medicines and herbs, most participants have relied on non-medical sources. Moreover, potential misuse and unnecessary use of certain drugs have been identified.

## Supporting information

S1 File The questionnaire (English and Bangla version). (PDF)

S2 File Study data. (SAV)
