# Supplementary material for: Behavioral preventive measures and the use of medicines and herbal products among the public in response to Covid-19 in Bangladesh: A cross-sectional study
Source: PLoS One. 2020 Dec 11;15(12):e0243706. doi: 10.1371/journal.pone.0243706 (PMC7732085; doi:10.1371/journal.pone.0243706)
Supplement: S1 File — (PDF) [file pone.0243706.s001.pdf]

Behavioral preventive measures and the use of medicines and herbal products among the public in response to Covid-19 in Bangladesh: A cross-sectional study

Questionnaire- English

1. What is your age? ______

2. What is your gender?
    a. Male
    b. Female

3. Where is your residence?
    a. Rural
    b. Urban

4. What is your level of education?
    a. SSC or below
    b. HSC
    c. Bachelor's degree
    d. Master's degree or above

5. Are you married?
    a. No
    b. Yes

6. Do you have any chronic disease (Diabetes, Heart disease, Asthma, Cancer, Liver/Kidney disease, etc.)?
    a. No
    b. Yes

7. How much are you afraid of Covid-19?
    a. Not afraid
    b. Slightly afraid
    c. Very afraid

8. Are you washing your hands more often than before?
    a. No
    b. Yes

9. Are you staying home more often than before?
    a. No
    b. Yes

10. Are you abstaining from visiting crowded places?
    a. No
    b. Yes

11. Do you use facemask when going outside?
    a. No
    b. Yes

12. Have there been any changes in your smoking habit?
    a. I never smoke
    b. There has not been any change in my smoking habit
    c. I am smoking less than before
    d. I am smoking more than before

13. Have you experienced any symptoms related to Covid-19 (fever, dry cough, difficulty breathing, tiredness, sore throat, etc.?)
    a. No
    b. Yes

If the answer to question 13 is No, move to BOX 1. If YES, move to BOX 2

BOX 1 (Answer if you have NOT experienced any symptoms related to Covid-19)

1. Have you taken any medicine as a preventive measure against Covid-19 infection /lower risk of covid-19 infection in the future?
   a. No
   b. Yes

   If yes, which medicine? ____

2. Have you taken any herbal food/product as a preventive measure against Covid-19 infection/lower risk of covid-19 infection in the future?
   a. No
   b. Yes

   If yes, which herbal product? ____

3. If you have taken any medicine or herbal food/product, what was the source of advice/information? (mark all that apply)
   a. Physician
   b. Retail pharmacist
   c. TV/ newspaper
   d. Facebook/twitter
   e. Internet (Google)
   f. Family/Friend/relative
   g. Self-knowledge
   h. Not applicable

BOX 2 (Answer if you have experienced any symptoms related to Covid-19)

1. If you have experienced any symptom (s) related to Covid-19, have you taken any medicine to manage that symptom (s)?
    a. No
    b. Yes

    If yes, which medicine? ____

2. Did you take any medicine **before** experiencing Covid-19 related symptoms as a preventive measure against Covid-19 infection?
    a. No
    b. Yes

    If yes, which medicine? ____

3. Did you take any herbal food/product before experiencing Covid-19 related symptoms as a preventive measure against Covid-19 infection?
    a. No
    b. Yes

    If yes, which herbal product? ____

4. If you have taken any medicine or herbal food/product, what was the source of advice/information? (mark all that apply)
    a. Physician
    b. Retail pharmacist
    c. TV/ newspaper
    d. Facebook/twitter
    e. Internet (Google)
    f. Family/Friend/relative
    g. Self-knowledge
    h. Not applicable

Questionnaire- Bangla

১) আপনার বয়স _________

২) আপনার লিঙ্গ-

 পুরুষ মহিলা

৩) আপনি কোন ধরণের এলাকায় বাস করেন?

 গ্রাম শহর

৪) আপনার শিক্ষাগত যোগ্যতা কী?

 এসএসসি বা তার নীচে এইচএসসি
 স্নাতক/ ব্যাচেলর ডিগ্রি স্নাতকোত্তর/ মাস্টার্স ডিগ্রি বা তার উপরে

৫) আপনি কি বিবাহিত?

 না হ্যাঁ

৬) আপনার কি কোনও দীর্ঘস্থায়ী/ক্রনিক রোগ রয়েছে (ডায়াবেটিস, হৃদরোগ, হাঁপানি, ক্যান্সার, লিভার/কিডনী রোগ ইত্যাদি)?

 না হ্যাঁ

৭) আপনি কোভিড-১৯ এর ব্যাপারে কতটুকু আতঙ্কিত?

 আতঙ্কিত নই
 কিছুটা আতঙ্কিত
 খুবই আতঙ্কিত

৮) আপনি কি আগের চেয়ে বেশি ঘন ঘন হাত ধুচ্ছেন?

 না হ্যাঁ

৯) আপনি কি আগের চেয়ে বেশি সময় বাড়ির ভেতর থাকছেন?

 না হ্যাঁ

১০) আপনি কি জনবহুল স্থানে যাওয়া থেকে বিরত থাকছেন?

□ না □ হ্যাঁ

১১) আপনি কি ঘরের বাইরে যাওয়ার সময় ফেস মাস্ক ব্যবহার করেন?

□ না □ হ্যাঁ

১২) আপনার ধূমপানের অভ্যাসে কি কোনও পরিবর্তন হয়েছে?

□ আমি কখনই ধূমপান করি না
□ আমি আগের মতোই ধূমপান করছি
□ আমি আগের চেয়ে কম ধূমপান করছি
□ আমি আগের চেয়ে বেশি ধূমপান করছি

১৩) আপনার শরীরে কোভিড-১৯ সম্পর্কিত কোনও লক্ষণ (জ্বর, শুকনো কাশি, শ্বাসকষ্ট, ক্লান্তি, গলা ব্যাথা বা অন্যান্য) কি ইতোমধ্যে দেখা দিয়েছে?

□ না □ হ্যাঁ

যদি ১৩ নম্বর প্রশ্নের উত্তর না হয়, তবে বক্স ১ পুরণ করুন, আর যদি হ্যাঁ হয়, তবে বক্স 2 পূরণ করুন।
(পরবর্তী পৃষ্ঠা)

বক্স ১ - আপনার শরীরে কোভিড-১৯ এর লক্ষণ দেখা *না* দিয়ে থাকলে পুরণ করুন

১) ভবিষ্যতে আপনার কোভিড-১৯ হওয়ার সম্ভাবনা / ঝুঁকি কমানোর জন্য আপনি কি কোন ওষুধ খেয়েছেন?

☐ না ☐ হ্যাঁ

যদি খেয়ে থাকেন, তাহলে ওষুধের নাম লিখুন

----

২) ভবিষ্যতে আপনার কোভিড-১৯ হওয়ার সম্ভাবনা / ঝুঁকি কমানোর জন্য আপনি কি কোনও ভেষজ/ হারবাল পণ্য খেয়েছেন?

☐ না ☐ হ্যাঁ

যদি খেয়ে থাকেন, তাহলে কি খেয়েছেন?

----

৩) আপনি যদি কোনও ওষুধ বা ভেষজ পণ্য গ্রহণ করে থাকেন তবে ওষুধ / ভেষজ পণ্য ব্যবহার সম্পর্কে পরামর্শের উৎস কী ছিল? (প্রযোজ্য সকল উত্তর চিহ্নিত করুন)

☐ ডাক্তার
☐ ফার্মাসিস্ট
☐ টিভি / সংবাদপত্র
☐ ফেসবুক / টুইটার
☐ ইন্টারনেট (গুগল)
☐ পরিবার / বন্ধু / আত্মীয়
☐ নিজস্ব জ্ঞান
☐ প্রযোজ্য নয়

বক্স ২- আপনার শরীরে কোভিড-১৯ এর লক্ষণ দেখা দিয়ে থাকলে পুরণ করুন

১) আপনার শরীরে কোভিড-১৯ এর লক্ষণ দেখা যাওয়ার পর সুস্থ হওয়ার জন্য কি আপনি কোন ওষুধ খেয়েছেন?

☐ না ☐ হ্যাঁ

যদি খেয়ে থাকেন, তাহলে ওষুধের নাম লিখুন - ______________________________________

২) আপনার শরীরে কোভিড-১৯ এর লক্ষণ দেখা যাওয়ার আগেই কি আপনি কোভিড-১৯ এর ঝুঁকি কমাতে কোন ওষুধ খেয়েছিলেন?

☐ না ☐ হ্যাঁ

যদি খেয়ে থাকেন, তাহলে ওষুধের নাম লিখুন - ______________________________________

৩) আপনার শরীরে কোভিড-১৯ এর লক্ষণ দেখা যাওয়ার আগেই আপনি কোভিড-১৯ এর ঝুঁকি কমাতে কোনও ভেষজ/ হারবাল পণ্য খেয়েছিলেন?

☐ না ☐ হ্যাঁ

যদি খেয়ে থাকেন, তাহলে কি খেয়েছেন? - ______________________________________

৪) আপনি যদি কোনও ওষুধ বা ভেষজ পণ্য গ্রহণ করে থাকেন তবে ওষুধ / ভেষজ পণ্য ব্যবহার সম্পর্কে পরামর্শের উৎস কী ছিল? (প্রযোজ্য সকল উত্তর চিহ্নিত করুন)

☐ ডাক্তার
☐ ফার্মাসিস্ট
☐ টিভি / সংবাদপত্র
☐ ফেসবুক / টুইটার
☐ ইন্টারনেট (গুগল)
☐ পরিবার / বন্ধু / আত্মীয়
☐ নিজস্ব জ্ঞান
☐ প্রযোজ্য নয়
